# Alterations in the Levels of Growth Factors in Adolescents with Major Depressive Disorder: A Longitudinal Study during the Treatment with Fluoxetine

**DOI:** 10.1155/2019/9130868

**Published:** 2019-11-19

**Authors:** Enrique Becerril-Villanueva, Gilberto Pérez-Sánchez, Samantha Alvarez-Herrera, Manuel Iván Girón-Pérez, Rodrigo Arreola, Carlos Cruz-Fuentes, Lino Palacios, Francisco R. de la Peña, Lenin Pavón

**Affiliations:** ^1^Laboratory of Psychoimmunology, National Institute of Psychiatry, “Ramón de la Fuente”, Calzada México-Xochimilco 101, Colonia San Lorenzo Huipulco, Tlalpan, 14370 Mexico City, Mexico; ^2^Universidad Autónoma de Nayarit, Laboratorio de Inmunotoxicología, Boulevard Tepic-Xalisco s/n, Cd de la Cultura Amado Nervo, C.P, 63000 Tepic, Nayarit, Mexico; ^3^Centro Nayarita de Innovación y Transferencia de Tecnología A.C. Laboratorio Nacional para la Investigación en Inocuidad Alimentaria-Unidad Nayarit, Calle Tres s/n. Cd Industrial, Tepic, Nayarit, Mexico; ^4^Psychiatric Genetics Department, Clinical Research Branch, National Institute of Psychiatry, “Ramón de la Fuente”, Calzada México-Xochimilco 101, Colonia San Lorenzo Huipulco, Tlalpan, 14370 Mexico City, Mexico; ^5^Adolescent Clinic, Clinical Services, National Institute of Psychiatry, “Ramón de la Fuente”, Calzada México-Xochimilco 101, Colonia San Lorenzo Huipulco, Tlalpan, 14370 Mexico City, Mexico

## Abstract

Major depressive disorder (MDD) has a prevalence of 5% in adolescents. Several studies have described the association between the inflammatory response and MDD, but little is known about the relationship between MDD and growth factors, such as IL-7, IL-9, IL-17A, VEGF, basic FGF, G-CSF, and GM-CSF. It must be appointed that there are scarce reports on growth factors in adolescents with MDD and even fewer with a clinical follow-up. In this work, we evaluated the levels of growth factors (IL-7, IL-9, IL-17A, VEGF, basic FGF, G-CSF, and GM-CSF) in MDD adolescents and the clinical follow-up during eight weeks of treatment with fluoxetine. *Methods*. All patients were diagnosed according to the DSM-IV-TR, and the severity of the symptoms was evaluated using the Hamilton Depression Rating Scale (HDRS). Growth factors IL-7, IL-9, IL-17A, VEGF, basic FGF, G-CSF, and GM-CSF were quantified by cytometric bead array using serum samples from 22 adolescents with MDD and 18 healthy volunteers. *Results*. All patients showed clinical improvement since the fourth week of pharmacological treatment according to the HDRS. Considerably higher levels of IL-7, IL-9, IL-17A, VEGF, basic FGF, G-CSF, and GM-CSF were detected in MDD adolescents as compared to healthy volunteers. A significant but temporal decrease was detected in basic FGF, G-CSF, and GM-CSF at week four of fluoxetine administration. *Conclusions*. To the best of our knowledge, this is the first report to show alterations in the levels of growth factors, such as IL-7, IL-9, IL-17A, VEGF, basic FGF, G-CSF, and GM-CSF in MDD adolescents during eight weeks of clinical follow-up. These disturbances might be involved in the physiopathology of MDD since such growth factors have been proven to participate in the neural development and correct functioning of the CNS; therefore, subtle alterations in it may contribute to MDD.

## 1. Introduction

Major depressive disorder (MDD) is one of the mood alterations with the highest occurrence in adolescence [1]. Its onset during this stage of life is an essential risk factor for suicide and represents the second most common cause of death among teenagers [2]. It has been estimated that 2.8 million adolescents between the ages of 12 and 17 have experienced a depressive episode. MDD causes social, familial, and educational deterioration, which leads to a high rate of disability among this population [3]. Although several works on MDD describe alterations in the levels of cytokines [4], chemokines [5], growth factors [6], neurotransmitters, and hormones, there are few reports with a clinical follow-up in adolescents [7, 8]. In a previous work, we reported inflammatory profiles that include IL-2, IFN-*γ*, IL-1*β*, TNF-*α*, IL-6, IL-12, and IL-15, as well as IL-4, IL-5, IL-13, IL-1Ra, and IL-10 cytokines in depressed adolescents [8].

Soluble inflammatory mediators are molecules that not only activate and promote an efficient immune response [9] but also regulate neural maintenance and development throughout life [10]. A particular group of proinflammatory cytokines is involved in the upkeep of this balance since they can act as both hematopoietic and neurotrophic growth factors [6]. Growth factors control embryonic and postnatal states, promoting the adequate functioning of the central nervous system (CNS),therefore, subtle changes in the secretion and expression patterns of these factors are relevant to the physiopathology of MDD. There are descriptions of alterations in cognitive, emotional, and motor processes associated with a decrease in the levels of BDNF, EGF/ErbB3, EPO, IGF, NGF, TGF-*β*, and VEGF in peripheral blood [6]. The pharmacological treatment with monoamine oxidase (MAO) inhibitors, selective serotonin reuptake inhibitors (SSRIs), tricyclic agents, and serotonin and noradrenaline reuptake inhibitors (SNRIs) restores the levels of BDNF, NGF, FGF, TGF-*β*, VEGF, and IGF-1, as well as the expression of EGF [11–16]. Together, these findings have led to the consideration of growth factors as possible biomarkers for the diagnosis and prognosis of MDD [6]. In this work, we show significant alterations in the serum levels of seven growth factors in adolescent MDD patients during 8 weeks of pharmacological treatment with an SSRI (fluoxetine).

## 2. Methods

### 2.1. Patients

The study was performed in the National Institute of Psychiatry “Ramón de la Fuente Muñiz” in Mexico City, Mexico. We recruited 336 adolescents with MDD from January 2006 to December 2008, and 22 of them participated in the study based on the inclusion criteria. The inclusion criteria included men and women aged between 14 and 19 years who met the diagnostic criteria for major depressive disorder (MDD) per the DSM-IV-TR and had a minimum baseline score on the HRSD ≥ 14 and no history of treatment for MDD with SSRIs and whose current episodes were moderate, lasting no more than two years. All the patients who were included agreed to participate in the study (INPRF-2035) and signed informed consent forms. The patients were recruited per the clinical follow-up in the INPRF-2035 research protocol, as approved by the ethics committee of the National Institute of Psychiatry “Ramón de la Fuente Muñiz,” Mexico.

### 2.2. Healthy Volunteers

Eighteen healthy volunteers (HVs) were recruited from the general population between January 2006 and December 2008. Clinical parameters of the HVs were within normal reference ranges (data not shown). The MINI (Mini-International Neuropsychiatric Interview) confirmed that the HVs did not suffer from any mental disorder and all had been free from any medication use for at least three weeks before blood sampling. The demographic data of patients and healthy volunteers are shown in Table 1.

### 2.3. Clinical Procedures

**Figure 1:**
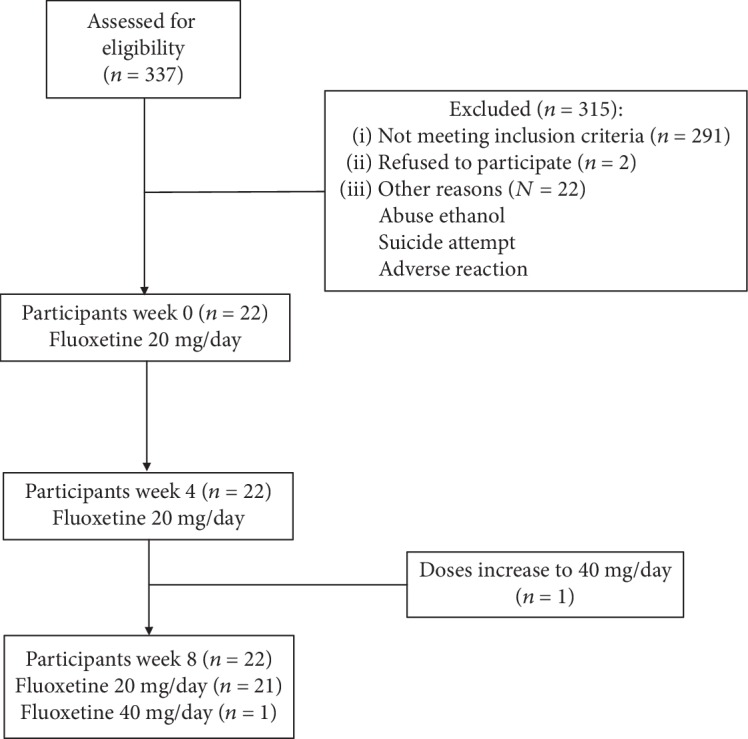
Flow diagram of eight-week fluoxetine treatment in adolescents with major depressive disorder.

Psychiatrists diagnosed all the subjects while the clinical status of adolescents with MDD was determined using the validated Spanish version of the 21-item Hamilton Depression Rating Scale (HDRS) [17]. Patients had not taken any antidepressants for at least 3 weeks prior to the study. After receiving a detailed explanation of the study aims, they signed written consent forms. All patients were given SSRIs. At the screening visit, after being administered with the HRSD, every subject underwent a laboratory examination to rule out any medical illnesses. All patients were evaluated monthly throughout the study by their psychiatrist, who applied the HRSD.  Figure 1 shows the total number of patients evaluated, the changes in their pharmacological treatment, and their reasons for withdrawal from the protocol.

### 2.4. Drugs

The dosage of fluoxetine was 20 mg/day. Doses were established for each patient by the psychiatrist and adjusted when necessary. All patients paid for their drugs out of pocket.

### 2.5. Serum Samples

Peripheral blood (10 mL) was collected by venipuncture from the cubital vein into Vacutainer® SST™ tubes with gel for serum separation (REF: 367988 BD Vacutainer System, Franklin Lakes, NJ, USA). Blood samples were centrifuged immediately (1.125 × g) at 4°C for 15 min to obtain serum. Serum samples were separated into aliquots and stored at –80°C until analysis.

### 2.6. Growth Factor Quantification

The levels of IL-7, IL-9, IL-17A, VEGF, basic FGF, G-CSF, and GM-CSF were measured in serum using a Bio-Plex Pro™ Human Cytokine 27-Plex Assay kit (Lot. #5029511) per the manufacturer's instructions. Analytes were detected using streptavidin phycoerythrin and quantified in a Bio-Plex MAGPIX™ Multiplex Reader (Bio-Rad Laboratories Inc., CA, USA). Analyte concentrations were calculated by interpolation using standard curves in Bio-Plex Manager™ (Bio-Rad Laboratories Inc., CA, USA; version 6.1). The ranges of detection were as follows (pg/mL): IL-7: 2.9–33,292; IL-9: 0.8–9,281; IL-17A: 2.4–28,099; VEGF: 2.6–29,672; basic FGF: 1.3–14,858; G-CSF: 2.4–28,053; and GM-CSF: 1.5–17,729.

### 2.7. Statistical Analysis

Statistical analysis for HDRS scores and growth factors was performed using GraphPad Prism, version 6.00, for MAC OS X (GraphPad Software, La Jolla, CA, USA). Homogeneity of variance and normality tests were applied, followed by a one-way ANOVA with Bonferroni's post hoc test. All values were expressed as mean ± standard deviation. Statistical significance was attributed when *P* < 0.05.

## 3. Results

### 3.1. Demographic Data

Demographic data of twenty-two adolescents with MDD and eighteen healthy volunteers are described in Table 1.

### 3.2. HDRS

Adolescents with MDD had an HDRS score of 19.41 ± 4.72 at the beginning of the study (W0), 9.13 ± 3.5 at week four (W4), and 6.09 ± 2.4 at week eight (W8). HDRS scores at W4 and W8 were significantly lower when compared to W0, as shown in Table 1.

### 3.3. Growth Factors

*(1) IL-7, IL-9, IL-17A, and VEGF*. Serum levels of IL-7, IL-9, IL-17A, and VEGF were significantly higher in adolescents with MDD at W0 compared with those in HVs (IL-7: *F* = 23.97; IL-9: *F* = 8.44; IL-17A: *F* = 13.10; and VEGF: *F* = 21.79; in all cases *df* = 80.3 and *P* < 0.0001). Levels were consistently elevated during the eight weeks of treatment (Table 1).

*(2) Basic FGF, G-CSF, and GM-CSF*. We observed a significant increase in serum levels of basic FGF, G-CSF, and GM-CSF in adolescents with MDD (basic FGF: *F* = 17.25; G-CSF: *F* = 20.24; and GM-CSF: *F* = 28.50; in all cases *df* = 80, 3 and *P* < 0.0001). Interestingly, basic FGF, G-CSF, and GM-CSF showed a significant decrease after four weeks of treatment with fluoxetine (W0 vs. W4; *P* < 0.01), but they increased again at W8 (Table 1).

## 4. Discussion

Our results show that adolescents with MDD had a clinical improvement from week four of treatment with fluoxetine, as reported in previous works in adolescents [8, 18]. However, we found no correlation between HDRS score and the levels of growth factors (data not shown).

### 4.1. IL-7

IL-7 is mainly associated with the development of T and B cells and is considered a necessary hematopoietic factor for the maintenance and proliferation of primary and secondary lymphoid organs [19]. Our results show significantly high levels of IL-7 in adolescents with MDD as reported in previous works [20, 21], but contrary to the report by Lehto et al. [22]. Comorbidities as sleep disorders, metabolic syndrome, number of cigarettes smoked, and daily alcohol consumption have been associated with decreased levels of IL-7 [22]. Unlike Dahl et al. [20], who reported a significant decrease of IL-7 after 12 weeks of treatment, our group found no changes in IL-7 levels during the clinical follow-up. It should be highlighted that the work of Dahl et al. was performed in adults who received dual antidepressant and SSRI treatment during 12 weeks [20]. It is known that IL-7 preferentially promotes a polarization towards Th-1, favoring a proinflammatory profile [23, 24], something common in MDD patients. Additionally, IL-7 is able to act as a neural growth factor by influencing the development of neurons at a molecular level, which in turn affects the brain architecture [25]. The link between IL-7 and neural development gains special interest when talking about MDD, and this work provides new evidence on alterations of IL-7 during the course of pharmacological treatment with fluoxetine, a topic little explored in adolescents.

### 4.2. IL-9

IL-9 is a Th-2 cytokine that has been associated with Th17 cells [26]. It has been widely described as a growth factor that regulates hematopoiesis, mast cell growth, and B cell development [26]. Although its role was described over 20 years ago, its direct participation in MDD is yet to be found, and there are no reports of its involvement in adolescents with MDD. Recently, Karlsson et al. detected a positive correlation between IL-9 and prenatal depression and anxiety [27] as well as a possible link between prenatal stress and the neonatal health. In addition, Shelton et al. found an increase in the expression of IL-9 in the frontal cortex of MDD patients [28], which suggests that the overexpression of IL-9 is associated with the severity of MDD. However, in our results, we did not find a correlation between IL-9 and HDRS score (data not shown).

### 4.3. IL-17A

IL-17A is a cytokine mainly produced by Th17 cells and is strongly associated with a series of inflammatory conditions and autoimmune diseases [29]. Moreover, it is known that IL-17 is able to stimulate angiogenesis and tumor growth [30]. In 2011, Chen et al. reported that patients with MDD coursing with an autoimmune process have high levels of IL-17 [31], but the role of IL-17 remains unclear in patients with MDD in the absence of an autoimmune disorder, as in our results. In adolescents with MDD, there is a correlation between anxiety symptoms and the levels of IL-17; moreover, the levels of this cytokine are not affected by the administration of antidepressants [7]. Our results show higher levels of IL-17 in adolescents with MDD as compared with healthy volunteers, as in previous reports in adults [7, 32], and the treatment with fluoxetine did not alter the levels of this growth factor. Interestingly, there is a study in vitro that reports that citalopram affects the levels of IL-17 in peripheral mononuclear cells from adult MDD patients [33]. Furthermore, IL-17 is mainly produced by Th17 cells, which have important roles in immune response in the CNS, as the regulation of the microglial activation. In fact, it has been suggested a role of both Th17 cells and IL-17 in the neuroprogression of MDD [34]. Moreover, in a mouse model, it was demonstrated that the administration of IL-17 antibodies was able to produce antidepressant-like effects [35], supporting the role of IL-17 in the development of MDD.

### 4.4. FGF

Fibroblast growth factor (FGF) is involved in important processes as long-term potentiation, neurogenesis, and proliferation of neural progenitor cells [36], which are all closely related to MDD. It is known that FGF is altered in adults with MDD, but little is known about it in adolescents. A detailed meta-analysis on FGF showed increased levels of FGF in adults with MDD [37], which agrees with our results in adolescents. On the contrary, He et al. reported lower levels in patients with MDD as well as a reduction of FGF after eight weeks of treatment with antidepressants [12]. In this sense, we also observed a reduction of FGF during treatment, but only in a temporal manner at the fourth week. Although the role of this growth factor is associated with neurogenesis and angiogenesis, more evidence is needed to demonstrate that the peripheral levels correlate to those in the CNS. Similarly, the lack of information regarding its role in the adolescent population represents an opportunity for future studies.

### 4.5. VEGF

The vascular endothelial growth factor (VEGF) plays an essential role in angiogenesis; therefore, it is considered to be a neurotrophic factor. Numerous studies in adults have shown significantly higher levels of VEGF in MDD patients versus healthy volunteers [38]. Within this frame, our results also show an increase in VEGF in adolescents with MDD but no affectations by the treatment with fluoxetine, which also agrees with previous reports [39].

### 4.6. G-CSF and GM-CSF

G-CSF and GM-CSF are two important hematopoietic factors that have been associated with MDD. These growth factors can promote macrophage migration from the bone marrow, giving rise to the inflammatory process observed in MDD. The link between macrophages and MDD is known as the *macrophage theory of depression* [40]. Our results in adolescents with MDD are consistent with those described in previous works in adults, which reported significantly higher levels of G-CSF and GM-CSF in depressed patients [20]. Moreover, Kiraly et al. observed a correlation between the high levels of GM-CSF in MDD patients and the subjects' resistance to antidepressants [41]. Finally, we also observed a significant but temporal reduction of G-CSF and GM-CSF at week four of treatment, as in previous reports [20].

Taken together, all these results demonstrate that GM-CSF and G-CSF may play crucial roles in MDD given that they can promote the mobilization of leukocytes from the bone marrow, leading to an increase in macrophages and the release of cytokines in inflammatory processes as depression. These findings reinforce the *macrophage theory of depression* in MDD onset [40] but now in adolescents.

## 5. Conclusions

Our results showed a significant increase in the circulatory levels of growth factors in adolescents with MDD in comparison with healthy volunteers (HV). This increase was consistent throughout the 8-week pharmacological treatment with fluoxetine [8]. To the best of our knowledge, this is the first report to show alterations in the levels of growth factors such as IL-7, IL-9, IL-17A, VEGF, basic FGF, G-CSF, and GM-CSF in adolescents with MDD during eight weeks of clinical follow-up. These disturbances could be involved in the physiopathology of MDD because it has been proven such growth factors participate in the neural development and correct functioning of the CNS; therefore, subtle alterations in it may contribute to MDD.

### 5.1. Limitations of This Study

The patient cohort was small, mainly due to the difficulties involved in pickup adolescents in clinical studies, but the numbers of HVs and patients were sufficient for statistical analysis. Also, the clinical follow-up was only 8 weeks long. In all cases, the discrepancies in our values and those by other groups can be explained by differences in samples (serum or plasma), detection techniques, sample size, age of participants, and confounding factors such as alcohol and tobacco consumption and body mass index.

## Figures and Tables

**Table 1a:** (a) Demographic data

	*Healthy volunteers(n* = 18)	*Patients(n* = 22)
*Demographics*		
Age (years)	18.9 ± 1.2	17.1 ± 2.3
Gender (male/female)	4/14	4/18
BMI (kg/m^2^)	23.2 ± 2.1	23.1 ± 2.1
Education (years)	12.9 ± 1.2	11.5 ± 2.6
Family history (yes/no)	3/15	8/14
First episode	NA	8
Recurrent episode	NA	14

**Table 1b:** (b) Molecular and clinimetric data

	*HV*	*Patients*			*Statistical post hoc analysis*		
	*n* = 18	*W0(n* = 22)	*W4(n* = 22)	*W8(n* = 22)	*W0 vs. HV*	*W4 vs. W0*	*W8 vs. W0*
*Cytokine serum levels (pg/mL)*							
IL-7	10.7 ± 1.8	23.7 ± 6.4	21.0 ± 5.5	24.3 ± 6.7	^∗∗∗∗^	ns	ns
IL-9	20.7 ± 3.9	30.3 ± 7.8	28.3 ± 9.8	32.7 ± 7.8	^∗∗^	ns	ns
IL-17A	348.4 ± 16.8	424.8 ± 48.5	391.1 ± 55.7	420.4 ± 36.0	^∗∗∗∗^	ns	ns
FGF basic	84.8 ± 6.5	116.7 ± 18.0	98.7 ± 21.2	115.8 ± 14.2	^∗∗∗∗^	^∗∗^	ns
VEGF	95.7 ± 15.2	150.7 ± 33.3	146.0 ± 24.5	145.1 ± 17.5	^∗∗∗∗^	ns	ns
G-CSF	232.9 ± 21.1	333.0 ± 54.5	285.3 ± 49.2	326.4 ± 45.1	^∗∗∗∗^	^∗∗^	ns
GM-CSF	28.3 ± 6.2	74.0 ± 17.5	57.0 ± 21.2	64.1 ± 14.2	^∗∗∗∗^	^∗∗^	ns
*Clinical psychiatric scale*							
HDRS	NA	19.41 ± 4.72	9.13 ± 3.5	6.09 ± 2.4	NA	^∗∗∗∗^	^∗∗∗∗^

Values are presented as mean ± standard deviation (SD). Statistical analysis was performed by one-way ANOVA with Bonferroni's post hoc. Statistical significance was attributed when ^∗∗^*P* < 0.01 and ^∗∗∗∗^*P* < 0.0001. HV = healthy volunteers; NA = not applicable; ns = not significant *P* > 0.05; vs. = versus; HDRS = Hamilton Depression Rating Scale; W = weeks of clinical follow-up.

## Data Availability

The cytokine levels and psychiatric values used to support the findings of this study are available from the corresponding author upon request.
